# Nuclear magnetic resonance (NMR)-based metabolome profile evaluation in dairy cows with and without displaced abomasum

**DOI:** 10.1080/01652176.2019.1707907

**Published:** 2020-01-07

**Authors:** Abdullah Basoglu, Nuri Baspinar, Leonardo Tenori, Cristina Licari, Erdem Gulersoy

**Affiliations:** a Department of Internal Medicine, Faculty of Veterinary Medicine, Selcuk University, Selcuklu, Konya, Turkey; b Department of Biochemistry, Faculty of Veterinary Medicine, Selcuk University, Selcuklu, Konya, Turkey; c Interuniversitary Consortium for Magnetic Resonance of Metalloproteins (C.I.R.M.M.P.), Sesto Fiorentino (Florence), Italy; d Magnetic Resonance Center (CERM), University of Florence, Sesto Fiorentino (FI), Italy

**Keywords:** Cattle, cow, metabolomics, NMR, displaced abomasum

## Abstract

**Background:**

Displaced abomasum (DA) is a condition of dairy cows that severely impacts animal welfare and causes huge economic losses.

**Objective:**

To assess the metabolic status of the disease using metabolomics in serum, urine and liver samples aimed at both water soluble and lipid soluble fractions.

**Methods:**

Fifty Holstein multiparous cows with DA (42 left, 8 right) and 20 clinically healthy Holstein multiparous cows were used. Left DA was associated with concomitant ketosis in 19 animals and right in two. NMR-based metabolomics approach and hematological and biochemical analyses were performed. Statistical analysis was carried out on ^1^H-NMR data after they have been normalized using PQN method.

**Results:**

Contrary to generated PCA score plots the OPLS-supervised method revealed differences between healthy animals and diseased ones based on serum water-soluble samples. While water and lipid soluble metabolites decreased in serum samples, fatty acid fractions and cholesterol were increased in liver samples in DA affected cows. The metabolomic and chemical profiles clearly revealed that cows with DA (especially with LDA) were at risk of ketosis and fatty liver. Serum hippuric acid concentration was significantly higher in healthy cows in comparison with LDA, whereas serum glycine concentration was reported higher for healthy when compared to RDA affected animals.

**Conclusion:**

A biochemical network and pathway mapping revealed ‘valine, leucine and isoleucine biosynthesis’ and ‘phenylalanine, tyrosine and tryptophan biosynthesis’ as the most probable altered metabolic pathway in DA condition. Serum was advocated as the optimal biological matrix for the ^1^H-NMR analysis.

## 1. Introduction

Following parturition, up to 35–50% of high producing dairy cows are affected by metabolic and infectious diseases. Displaced abomasum is a multifactorial disorder usually diagnosed in early lactation dairy cows, and is a common inherited condition in Holstein cows (Zerbin et al. 2015; Doll 2015; Caixeta et al. 2018). The incidence of DA in the United States dairy herds was determined to be approximately 3.5% (NAHMS-USDA 2017). Economic analyses have determined that the average cost per DA diagnosis is more than $700 when accounting for direct and indirect costs (McArt et al. 2015).

Understanding DA in cattle has been the objective of numerous in vitro and in vivo studies. However, a complete elucidation of its pathogenesis has still to be achieved (Sickinger 2017). Proteomics and metabolomics have emerged as valuable techniques to characterize proteins and metabolite assets from tissue and biological fluids, such as milk, blood, and urine (Takis et al. 2018). There is a multitude of studies about the metabolic backgrounds of such so-called production diseases like ketosis, fatty liver, or hypocalcemia, although the investigations aiming to assess the complexity of the pathophysiological reactions are largely focused on gene expression, that is, transcriptomics. For extending the knowledge toward the proteome and the metabolome, the respective technologies are of increasing importance (Vignoli et al. 2019) and can provide an overall view of how dairy cows react to metabolic stress, which is needed for an in-depth understanding of the molecular mechanisms of the related diseases (Ceciliani et al. 2018). Displaced abomasum occurs simultaneously with fatty liver, but the relationship between the diseases are not clear (Ingvartsen 2006).

The aim of this study was to reveal new potential biomarkers representing the metabolic status of DA by using NMR-based metabolome profile evaluation and providing possible clues into the pathogenic mechanisms for DA.

## 2. Materials and methods

The experimental design was approved by the Committee on Use of Animals in Research of the Selcuk University, Faculty of Veterinary Medicine (Protocol No. 68/2017).

### 2.1. Animals

Fifty with displaced abomasum (42 LDA and 8 RDA), and 20 healthy Holstein multiparous cows within 1 month of parturition were used as animal material (Table 1). Dietary composition and nutrient level daily for the diseased animals were as follows: corn silage 12 kg, sugar beet pulp 10 kg, wheat straw 4.5 kg and concentrate 8.5 kg. The concentrate was consisted of 35% barley, 19.85% wheat, 15% wheat bran, 25% cotton seed meal, 3% limestone, 0.3% salt, and 0.35% vitamin–mineral mixture. It will be contained 21.5% crude protein and 2,850 kcal/kg metabolizable energy.

**Table 1.** Characterizations of the Holstein cows (±SD).

Parameters	Cow groups		
	Healthy	LDA	RDA
Postpartum days	13 ± 5	14 ± 6	15 ± 6
Age (yr)	3 ± 1	3 ± 1	3 ± 1
Parity	2 ± 1	2 ± 1	2 ± 1
Milk yield (kg/d)	24.82 ± 7.55	24.07 ± 7.38	23.00 ± 1.68
Milk fat %	3.55 ± 1	3.65 ± 1	3.45 ± 1
Milk protein %	3.06 ± 1	3.0 ± 1	3.05 ± 1

Displacement diagnosis was based on the history, the presence of the characteristic ping on simultaneous auscultation and percussion and exclusion of other causes of left- or right-sided pings. Ultrasonography was performed in confirming a diagnosis of LDA and RDA. Among cows with LDA, 19 were associated with concomitant ketosis. Two cows with RDA had concomitant ketosis too. Ketosis was detected by urine chemistry analyzer using urine test strips (Bayer Clinitek 50, Leverkusen, Germany) and blood ketone meter using blood ketone test strips Abbott Optium Xceed Pro, Oxford, UK). A Liptak test-needle was placed in the viscus to remove fluid, and pH was measured when needed (Guard 1990). All diagnoses were confirmed during a surgical operation. Control animals were also multiparous within 1 month of lactation and chosen via the same clinical and hematological methods.

### 2.2. Blood, urine and liver sampling

Blood samples were collected from the coccygeal vein into heparin and K-EDTA coated tubes. Blood samples were immediately used for hematological measurements. Serum samples harvested within an hour by centrifugation for 15 min at 3000 rpm were stored at −80 °C until analysis. Urine samples were obtained via a sterile, rigid metal catheter (approximately 0.5 cm in diameter and 40 cm long). Liver biopsies were collected on the right side, proximal within the 11th or 12th intercostal space using a Tru-cut biopsy trocar (Merit Medical, Maastricht, The Netherlands).

Serum, urine, and liver samples were thawed on ice and extracted using a dual methanol-chloroform extraction (for protein precipitation and separation of hydrophilic and lipophilic fractions) as previously described (Serkova et al. 2005). This eliminates macromolecules (e.g., proteins) and establishes a fused metabolic profile for water-soluble and lipid-soluble metabolites. Briefly, 0.5 ml of ice-cold serum or urine (0.5 mg of the liver) was mixed with 0.5 ml of chloroform: methanol (1:1 vol/vol) (0.1 ml of chloroform:0.2 ml of methanol and 0.04 ml of distilled water for liver) and centrifuged. The supernatant (organic phase) was collected, and the pellet was resuspended with 0.5 ml of chloroform/methanol (0.2 ml of chloroform/methanol for liver) and centrifuged. The supernatants were combined, and 0.5 ml of ice-cold water (0.1 ml of ice-cold water for liver) was added to ‘wash out’ remaining water-soluble metabolites from the organic phase. After 15 min at −20 °C, the upper (aqueous) phase was removed and added to the remaining pellet (to wash out remaining water-soluble metabolites from the pellet), 1 ml of water was added, and the sample was centrifuged and lyophilized by evaporation at 42 °C.

### 2.3. Hematological analysis

Hematological analyses including complete blood count (erythrocytes and leukocytes counts, MCV, MCHC, PCV, and Hb) were performed by automated cell counter (MS4e, Melet Schloesing Laboratories, Osny, France) and blood gas analysis (pH, pCO_2_, pO_2_, HCO_3_ and BE) by blood gas analyzer (Gem Premier 3000, Instrumentation Laboratory, Bedford, MA 01730, USA).

### 2.4. Chemical analysis

Serum was harvested within 1 h by centrifugation for 15 min at 3000 rpm, and stored at –80 °C until analysis. It was analyzed for glucose, lactate, cholesterol, triglyceride, total protein, albumin, total bilirubin, BUN, creatinine, some minerals (Na, Mg, P, K, Ca and ionized Ca); some enzyme activities (AST, GGT, LDH, and CPK), NEFA, and CRP by spectrometry (Autoanalyzer/BT3000 Plus, Rome, Italy). Blood BHBA levels were measured from whole blood by using Freestyle Optium H B-ketone, Abbott ® test strips (Allschwil, Switzerland).

### 2.5. Samples preparation for ^1^H-NMR spectroscopy

Dried water-soluble samples were dissolved in 700 µL of ^2^H_2_O (99.9 atom % D, Sigma Aldrich, St. Louis, Missouri, USA) and homogenized by vortexing for 1 min. Then, they were centrifuged (3000 rpm at 4 °C for 15 min) and 630 µL of each supernatant was added to 70 µL of potassium phosphate buffer (1.5 M K_2_HPO_4_, 100% (v/v) ^2^H_2_O, 2 mM NaN_3_, 5.8 mM deuterated trimethylsilyl propanoic acid (TMSP); pH 7.4). After stirring, a total of 600 µL from each mixture was transferred into 5 mm NMR tubes (Bruker BioSpin s.r.l) for the analysis.

The dried lipid extracts were dissolved in 700 µL of CDCl_3_ (99.8 atom % D, Sigma Aldrich) and homogenized by vortexing for 1 min. An aliquot of 600 µL from each sample was transferred into 5 mm NMR tubes (Bruker BioSpin s.r.l) for the analysis.

### 2.6. NMR analysis

For each sample one-dimensional ^1^H-NMR spectra were acquired using a Bruker 600 MHz spectrometer (Bruker BioSpin s.r.l; Rheinstetten, Germany) operating at 600.13 MHz proton Larmor frequency and equipped with a 5 mm PATXI ^1^H-^13^C-^15^N and ^2^H-decoupling probe including *a*–*z* axis gradient coil, an automatic tuning and matching (ATM), and an automatic and refrigerated sample changer (SampleJet, Bruker BioSpin s.r.l; Darmstadt, Germany). For temperature stabilization, a BTO 2000 thermocouple was used at the level of approximately 0.1 K at the sample. Before starting the measurement, samples were kept for at least 5 minutes inside the NMR probe head to equilibrate temperature, at 300 K for urine water soluble samples and at 310 K for all the other extracts.

For each water and lipid-soluble sample, a one-dimensional ^1^H NMR spectrum was acquired using a standard Nuclear Overhauser Effect Spectroscopy pulse sequence (NOESY 1Dpresat; noesygppr1d.com; Bruker BioSpin) using 98,304 data points, a spectral width of 18,028 Hz, an acquisition time of 2.7 s, a relaxation delay of 4 s, a mixing time of 0.01 s and a different number of scans according to the type of extract (128 scans for both serum water/lipid soluble and liver-lipid soluble samples; 256 scans for liver water-soluble extracts; 64 scans for both urine water and lipid soluble samples).

In addition, for serum water-soluble extracts, another ^1^H-NMR spectrum was acquired using a standard spin echo Carr-Purcell-Meiboom-Gill pulse sequence (CPMG) (Meiboom and Gill 1958) (cpmgpr1d.comp; Bruker BioSpin) with 128 scans, 73,728 data points, a spectral width of 12,019 Hz an acquisition time of 3.1 s, a relaxation delays of 4 s and a total spin-echo delay of 80 ms.

### 2.7. Spectral processing

Before applying Fourier transform, free induction decays were multiplied by an exponential function equivalent to 0.3 Hz line-broadening factor. Transformed spectra were automatically corrected for phase and baseline distortions and through TopSpin 3.2 (Bruker BioSpin software), they were calibrated using the anomeric glucose doublet at 5.24 ppm for serum water-soluble extracts, the TMSP singlet at 0.00 ppm for hydrophilic extracts of liver and urine and the chloroform singlet at 7.20 ppm for lipid soluble samples.

Using the bucketing procedure each 1D spectrum, in the range of 0.2 and 10.0 ppm, was segmented into 0.02 ppm chemical shift bins and the corresponding spectral areas were integrated using AMIX software (version 3.8.4, Bruker BioSpin). Through this technique, the number of total variables is reduced and small shifts in the signals are compensated, making the analysis more robust and reproducible (Holmes et al. 1994).

For water-soluble extracts, regions between 4.60 and 4.85 ppm containing residual H_2_O signal were removed, instead, regions between 6.90 and 7.55 ppm containing chloroform signal were excluded from spectra of lipid-soluble extracts.

On the remaining bins, probabilistic quotient normalization (PQN; Dieterle et al. 2006) was applied prior to pattern recognition.

Of note, systemic biofluids, that is, urine and serum, are able to reflect the global response of an organism to a disease status contrary to tissue samples. They require a simple and minimally invasive collection (Vignoli et al. 2019). Therefore, both urine and serum represent optimal biological matrices for the ^1^H-NMR analysis. However it should be realized that urine metabolites are more influenced by factors like age, diet, gut microbiota and/or other pathophysiological stimuli. Thus, urine ^1^H-NMR spectra are often more variable and contain crowded regions with a lot of signal overlaps and shifts, while serum samples, being more simple to be analyzed, could actually find a practical veterinary use.

### 2.8. Statistical analysis

Statistical significance was determined with one way ANOVA test for all the hematological and biochemical variables.

All metabolomic analysis was performed using R, an open source software for statistical analysis of data (Ihaka and Gentleman 1996). Multivariate analysis was applied on processed data and Principal Component Analysis (PCA) was used as a first exploratory approach. Orthogonal projections to latent structures (OPLS) analysis was applied as a supervised technique. In general, OPLS is a multivariate projection method which is normally used for modeling spectroscopic data. This algorithm is a modification of the PLS method (Wold 1998) and it is based on the idea to separate “response linearly related” and “response unrelated (orthogonal)” in data, providing a simpler method for interpreting them (Trygg and Wold 2002).

Accuracies and confusion matrices for different classifications were assessed by means of 100 cycles of Monte Carlo cross-validation scheme (MCCV, R script in-house developed). For this method, at each iteration, 90% of data are randomly chosen as a training set to build the model. The remaining 10% of data was tested and the accuracy for the classification was established. This procedure is repeated 100 times to derive an average discrimination accuracy for each group of subjects.

Univariate statistical analysis was carried on ^1^H-NMR data after they have been normalized using PQN method. In particular, spectral regions related to the different metabolites were assigned by using AMIX 3.8.4 (a Bruker BioSpin software) and published literature data. The same regions were integrated to get concentration values of metabolites in arbitrary units. Resulting values were used to determine discriminating metabolites among the groups of cows. Wilcoxon signed-rank test (Neuhäuser 2011) was applied to deduce metabolite differences among groups on the biological assumption that metabolite concentrations are not normally distributed. False discovery rate correction was used applying the Benjamini-Hochberg method (FDR) (Benjamini and Hochberg 1995) and the adjusted *p* value <0.05 was considered statistically significant.

Biochemical network mapping and related pathway analysis were also performed for serum metabolites (see the Supplementary Material file for details).

## 3. Results

### 3.1. Blood and chemistry profile

The main differences in biochemical parameters of LDA group included increased NEFA (*p* < 0.001), BHBA (*p* < 0.001) and triglyceride (*p* < 0.001) as compared to controls. There were significant increases in lactate, BE and CRP in RDA levels, and decreased K in RDA affected animals group. In spite of significant changes in other biochemical, and hematological parameters, they were within normal reference ranges (Tables 2 and 3).

**Table 2.** Hematological analysis (±SD).

Parameters	Cow groups		
	Healthy	LDA	RDA
Leukocytes (× 10^9^/L)	13.71 ± 4.81	16.70 ± 11.05	11.52 ± 3.21
Erythrocytes (× 10^12^/L)	7.35 ± 1.40^b^	8.36 ± 1.22^ab^	8.79 ± 1.29^a^
MCV	47.41 ± 5.73	48.15 ± 4.74	46.48 ± 6.04
MCHC (× 10 g/L)	34.62 ± 2.77^a^	31.64 ± 2.47^b^	30.73 ± 1.96^b^
Hematocrit (× 10^–2^ L/L)	34.15 ± 4.51^b^	40.11 ± 6.61^a^	41.05 ± 8.51^a^
Hemoglobin (× 10 g/L)	11.77 ± 1.28	12.75 ± 1.95	12.53 ± 2.22
Blood pH	7.44 ± 0.03^ab^	7.42 ± 0.07^b^	7.49 ± 0.06^a^
pO_2_ (mmHg)	35.72 ± 6.89	31.80 ± 5.05	31.87 ± 2.98
pCO_2_ (mmHg)	36.70 ± 5.80^b^	38.67 ± 5.68^b^	44.43 ± 3.92^a^
Bicarbonate (µmol/L)	25.30 ± 2.14^b^	24.88 ± 5.21^b^	33.47 ± 7.16^a^
Base excess (µmol/L)	1.17 ± 2.41^b^	1.10 ± 6.13^b^	10.18 ± 7.00^a^

^a, b, ab^ Means within a row with different superscripts differ (*p* < 0.05).

MCV = Mean corpuscular volume, MCHC = Mean corpuscular hemoglobin concentration.

**Table 3.** Biochemical analyses (±SD).

Parameters	Cow groups		
	Healthy	LDA	RDA
Na (mmol/L)	146.90 ± 5.41^a^	139.76 ± 5.30^b^	139.75 ± 5.20^b^
Cl (mmol/L)	104.60 ± 3.25^a^	98.30 ± 7.67^a^	85.25 ± 13.93^b^
K (mmol/L)	4.24 ± 0.38^a^	3.43 ± 0.64^b^	2.92 ± 0.73^b^
P (mmol/L)	2.3256 ± 0.42313^a^	1.59239 ± 0.54587^b^	1.9057 ± 0.9108^ab^
Mg (mmol/L)	0.8877 ± 0.17673^a^	0.63705 ± 0.17673^b^	0.8918 ± 0.3945^a^
Ca (mmol/L)	3.2625 ± 0.275^a^	2.7375 ± 0.33^b^	2.63 ± 0.395^b^
GGT (U/L)	15.90 ± 5.87^b^	41.52 ± 34.26^ab^	70.75 ± 63.33^b^
CK (U/L)	326.10 ± 212.07	487.64 ± 498.11	395.37 ± 279.31
AST (U/L)	134.40 ± 45.56	191.38 ± 120.86	175.62 ± 137.23
ALP (U/L)	58.30 ± 12.97	63.47 ± 29.59	79.62 ± 41.76
LDH (U/L)	2070.60 ± 247.84	2005.59 ± 571.85	2109.75 ± 381.83
BUN (mmol)	2.96 ± 0.6^b^	3.64 ± 2.19^b^	7.81 ± 5.85^a^
Creatinine (μmol/L)	96.36 ± 13.26^b^	101.66 ± 33.59^b^	143.21 ± 79.56^a^
Total biluribin (μmol/L)	37.28 ± 18.1^a^	12.9 ± 6.8^b^	12.9 ± 7.0^b^
Glucose (mmol/L)	2.93 ± 0.54^b^	4.59 ± 2.84^b^	7.06 ± 3.12^a^
Lactate (mmol/L)	1.49 ± 1.01^b^	2.80 ± 2.39^ab^	3.97 ± 3.99^a^
Cholesterol (mmol/L)	3.390 ± 1.5^6^	2.3 ± 1.04	2.86 ± 1.21
Triglyceride (mmol/L)	0.35 ± 0.34^ab^	0.63 ± 0.51^a^	0.24 ± 0.16^b^
Total protein (g/L)	0.08 ± 0.01^a^	0.07 ± 0.01^b^	0.07 ± 0.01^b^
Albumin (g/L)	0.036 ± 0.00^a^	0.0324 ± 0.01^ab^	0.03 ± 0.00^b^
NEFA (mmol/L)	0.28 ± 0.36^b^	1.26 ± 0.60^a^	1.09 ± 0.56^a^
BHBA (mmol/L)	0.82 ± 0.50^b^	1.94 ± 1.35^a^	0.97 ± 0.77^ab^
CRP (nmol/L)	5.52 ± 4.57^a^	10 ± 5.81^ab^	12.67 ± 6.38^b^

^a, b, ab^ Means within a row with different superscripts differ (*p* < 0.05).

NEFA = Non-esterified fatty acid, BHBA = β-hydroxybutirate, CRP = C-reactive protein.

### 3.2. NMR-Based metabolomic evaluation

NMR spectra of serum, liver and urine samples were acquired. Two samples for serum water-soluble extracts; five for serum lipid extracts; six for liver lipid soluble samples and five for urine lipid soluble samples were removed from the statistical analysis because of the bad quality of the generated spectra.

**Figure 1.**
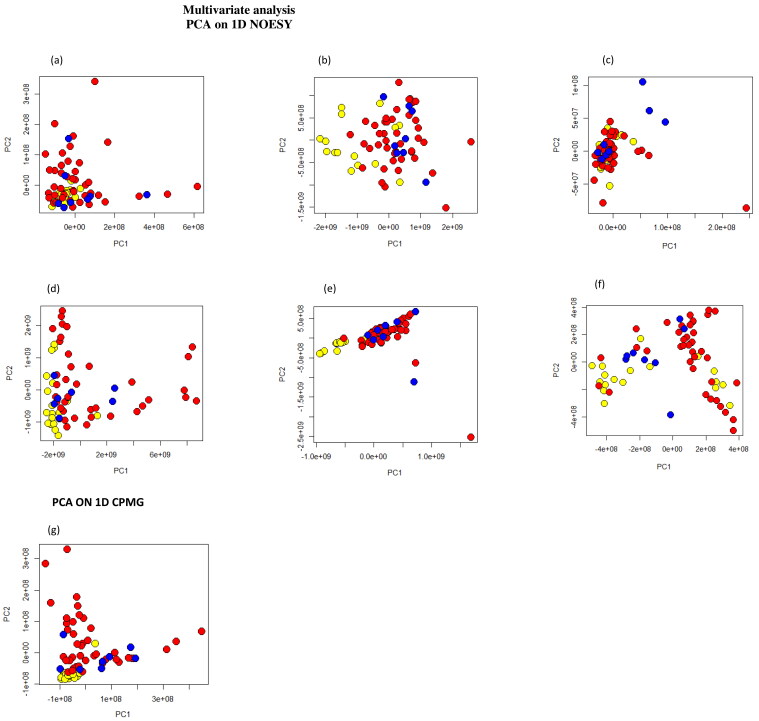
Principal component analysis (PCA) score plots. Each dot represents a single ^1^H-NMR spectrum and each color represents a different group of cows: healthy (yellows dots), LDA (red dots) and RDA (blue dots). (a) PCA on 1D NOESY spectra of serum water soluble samples; (b) PCA on 1D NOESY spectra of serum lipid soluble samples; (c) PCA on 1D NOESY spectra of liver water soluble samples; (d) PCA on 1D NOESY spectra of liver lipid soluble samples; (e) PCA on 1D NOESY spectra of urine water soluble samples; (f) PCA on 1D NOESY spectra of urine lipid soluble samples; (g) PCA on 1D CPMG spectra of serum water soluble samples.

Processed 1D spectra from all type of extracts including the three different groups of animals (healthy cows, cows with LDA and cows with RDA have been analyzed firstly using PCA to have an overview of the main differences between healthy cows and those with the displacement of the abomasum. Figure 1a–g shows the PCA score plots on 1D NOESY of all types of extracts and on 1D CPMG of serum water soluble samples.

The resulting score plots are not sufficient to discriminate healthy animals from cows with abomasum displacement and no evident differences are reported for the discrimination between cows with left or right displacement.

**Figure 2.**
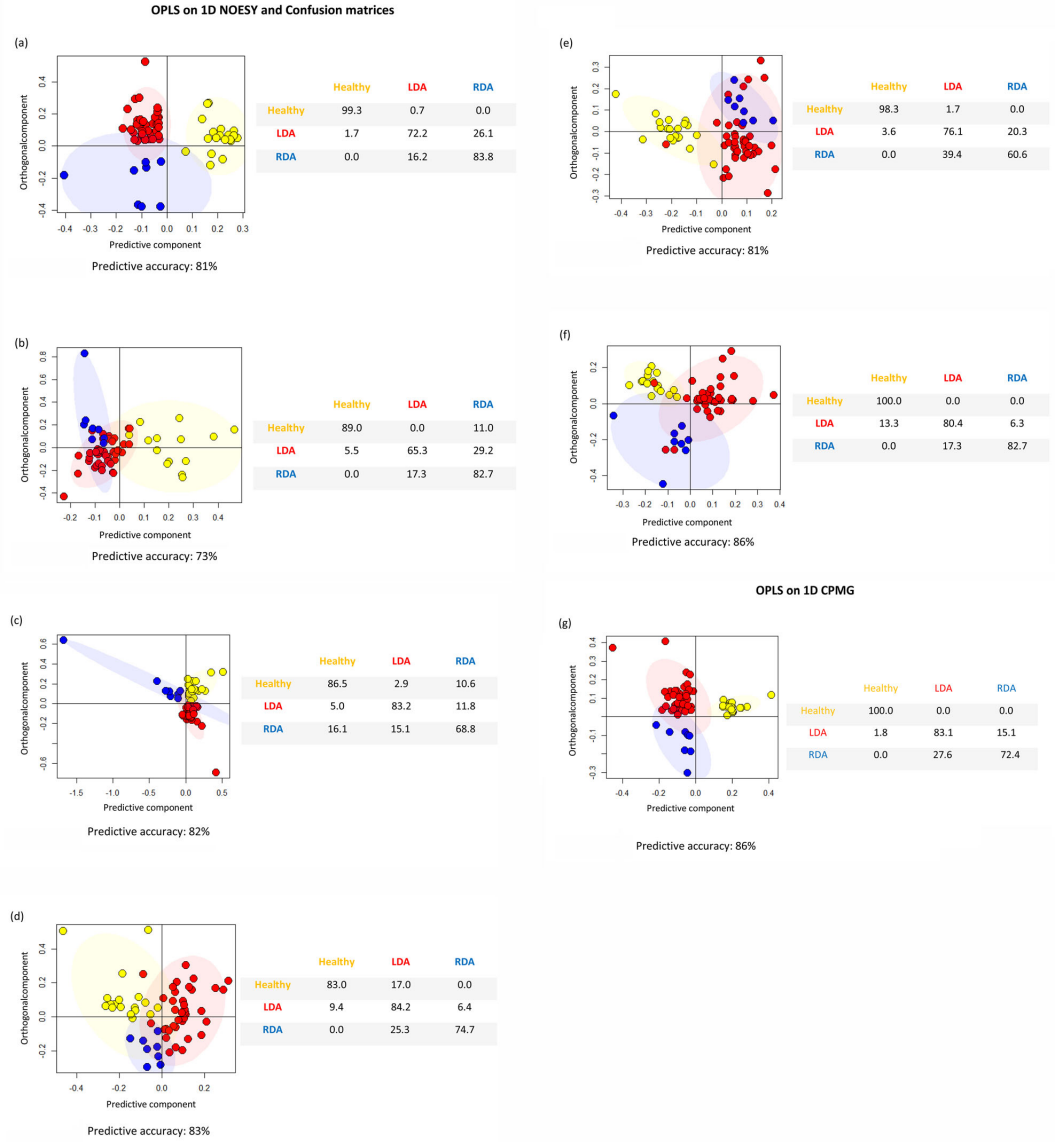
OPLS score plots of all type of extract. Each dot represents a single ^1^H-NMR spectrum and each color represents a different group of cows: healthy (yellows dots), LDA (red dots) and RDA (blue dots). (a) OPLS on 1D NOESY spectra of serum water soluble samples; (b) OPLS on 1D NOESY spectra of serum lipid soluble samples; (c) OPLS on 1D NOESY spectra of liver water soluble samples; (d) OPLS on 1D NOESY spectra of liver lipid soluble samples; (e) OPLS on 1D NOESY spectra of urine water soluble samples; (f) OPLS on 1D NOESY spectra of urine lipid soluble samples; (g) OPLS on 1D CPMG spectra of serum water soluble samples. Confusion matrices and related predictive accuracy of cross-validation analysis are reported for each model.

To explore differences between healthy and diseased cows, the OPLS-supervised method was used. OPLS models were built on 1D NOESY spectra and on 1D CPMG spectra for serum water soluble samples (Figure 2a–g) and a different number of components were retained in the model depending on the type of samples. All models comparing healthy versus diseased animals, as shown by prediction accuracies of cross-validation analyses in Figure 2a–g, are able to discriminate each group of cows with high accuracies and in particular, healthy animals were discriminated from the diseased ones with higher accuracies than 80% regardless of the type of sample under study. However, systemic biofluids, that is, urine and blood serum, are able to reflect the global response of an organism to a disease status with respect to tissues biopsies. They required a simple and minimally invasive collection (Vignoli et al. 2019). Therefore, both urine and blood serum represent optimal biological matrices for the ^1^H-NMR analysis of the DA disease, however urine metabolites are more influenced by, for example, age, lifestyle, diet, the activity of gut microflora or another symbiotic organism and/or other pathophysiological stimuli. Thus, urine ^1^H-NMR spectra are often more variable and contain crowded regions with a lot of signal overlaps and shifts, while serum blood samples, being more simple to be analyzed, could actually find a practical veterinary use.

^1^H-NMR spectra were also analyzed to identify which metabolites are altered in the three groups of cows. The complete list of identified and quantified metabolites from both the lipophilic and the hydrophilic fractions from each type of sample is in Table 4; adjusted *p* values are reported only for metabolites that differ significantly (*p* value <0.05) among the various comparisons performed. In particular, for each type of sample, healthy cows were compared to subjects with both LDA and RDA. To highlight metabolites that are significantly different between the two types of displacements, the comparison between cows with left and right abomasum dislocation was performed too.

**Table 4.** Concentrations in arbitrary units (median ± Median Absolute Deviation (MAD)) of the metabolites assigned in serum, liver and urine samples (both hydrophilic and lipophilic fractions).

	Metabolites	Healthy (arbitrary units)	LDA (arbitrary units)	RDA (arbitrary units)	*“adjusted” p* value (<0.05)
**Serum water soluble (SWS)**	2-aminobutyrate	145.0 ± 63.4	67.9 ± 28.5	20.6 ± 10.1	<0.05 (healthy versus LDA) <0.001 (healthy versus RDA) <0.001 (LDA versus RDA)
	2-hydroxybutyrate	3.2 ± 3.2	201.2 ± 155.3	311.4 ± 165.4	<0.0000001 (healthy versus LDA) <0.001 (healthy versus RDA)
	3-hydroxybutyrate	4896.0 ± 984.3	8454.3 ± 5252.8	3221.2 ± 810.1	<0.05 (healthy versus LDA)
	3-hydroxyisobutyrate	212.3 ± 58.0	228.9 ± 68.2	265.9 ± 101.9	
	Acetate	15866.6 ± 2143.8	0.01 ± 0.01	0.01 ± 0.01	<0.00001 (healthy versus LDA) <0.05 (healthy versus RDA)
	Alanine	1603.0 ± 305.1	1082.9 ± 337.3	800.8 ± 230.0	<0.001 (healthy versus LDA) <0.0001 (healthy versus RDA)
	Benzoate	27.2 ± 14.8	18.5 ± 18.5	150.2 ± 130.7	
	Carnitine	681.9 ± 97.0	1000.0 ± 451.8	752.3 ± 354.5	<0.05 (healthy versus LDA)
	Citrate	2814.4 ± 242.9	698.6 ± 333.4	907.5 ± 188.2	<0.00000001 (healthy versus LDA) <0.0001 (healthy versus RDA)
	Choline	456.2 ± 86.6	531.9 ± 146.7	393.5 ± 110.8	
	Creatine	2285.0 ± 357.1	1788.7 ± 385.9	1518.3 ± 703.9	<0.05 (healthy versus LDA)
	Creatinine	943.9 ± 139.0	819.1 ± 172.9	1087.8 ± 449.4	
	Dimethyl sulfone	661.9 ± 189.9	179.7 ± 82.8	136.3 ± 41.3	<0.000000001 (healthy versus LDA) <0.00001 (healthy versus RDA)
	formate	650.8 ± 77.0	509.9 ± 155.6	564.1 ± 97.1	<0.05 (healthy versus LDA)
	Fructose	675.5 ± 110.5	745.0 ± 244.7	1466.1 ± 378.3	<0.05 (healthy versus LDA) <0.05 (healthy versus RDA)
	Glycine	2180.8 ± 213.5	1270.3 ± 460.7	184.8 ± 184.8	<0.05 (healthy versus LDA) <0.05 (healthy versus RDA) <0.05 (LDA versus RDA)
	Glucose	1233.0 ± 106.3	1105.7 ± 256.1	1434.0 ± 342.2	
	Glutamine	19.3 ± 7.8	40.1 ± 13.7	15.7 ± 13.2	<0.001 (healthy versus LDA)
	Hippurate	437.2 ± 115.7	58.2 ± 33.2	54.0 ± 15.8	<0.00000001 (healthy versus LDA) <0.00001 (healthy versus RDA)
	inosine	6.13 ± 6.13	25.1 ± 18.8	38.3 ± 10.1	<0.001 (healthy versus LDA) <0.05 (healthy versus RDA)
	Isoleucine	675.8 ± 106.4	352.1 ± 118.2	150.2 ± 50.3	<0.00001 (healthy versus LDA) <0.05 (healthy versus RDA)
	lactate	2240.6 ± 426.1	3023.0 ± 1328.1	3024.6 ± 900.6	<0.05 (healthy versus LDA) <0.05 (healthy versus RDA)
	leucine	1169.5 ± 195.8	816.5 ± 318.1	481.4 ± 136.5	<0.05 (healthy versus LDA)
	Lysine	559.9 ± 44.9	545.9 ± 58.3	468.9 ± 38.1	<0.05 (healthy versus RDA) <0.05 (LDA versus RDA)
	Mannose	205.0 ± 40.9	265.0 ± 72.5	457.2 ± 68.2	<0.001 (healthy versus LDA) <0.05 (healthy versus RDA)
	Myoinositol	83.7 ± 17.4	83.7 ± 24.0	93.9 ± 43.6	
	Phenylalanine	486.9 ± 60.0	342.7 ± 79.3	297.2 ± 98.2	<0.001 (healthy versus LDA)
	Proline	176.9 ± 27.8	146.0 ± 35.4	139.4 ± 61.5	<0.05 (healthy versus LDA)
	Proprionate	60.3 ± 16.0	27.6 ± 13.0	24.7 ± 8.6	<0.00001 (healthy versus LDA) <0.001 (healthy versus RDA)
	Succinate	791.9 ± 142.8	570.7 ± 170.8	558.5 ± 194.9	<0.05 (healthy versus LDA)
	Threonine	57.1 ± 10.1	47.0 ± 13.0	31.5 ± 10.4	<0.05 (healthy versus LDA) <0.05 (healthy versus RDA)
	Trimethylamine N-oxide	4120.1 ± 1074.2	1263.6 ± 340.4	1535.7 ± 281.8	<0.000000001 (healthy versus LDA) <0.0001 (healthy versus RDA)
	Tyrosine	210.2 ± 39.3	79.7 ± 26.5	65.7 ± 12.4	<0.0000000001 (healthy versus LDA) <0.0001 (healthy versus RDA)
	Unknow	806.8 ± 101.5	724.0 ± 210.9	755.7 ± 373.1	
	Valine	1037.2 ± 222.9	798.0 ± 267.0	360.8 ± 116.9	<0.05 (healthy versus LDA)
**Serum lipid soluble (SLS)**	Cholesterol C(18)H_3_	92228.9 ± 23815.5	47223.8 ± 13086.6	51198.9 ± 8451.9	<0.00001 (healthy versus LDA) <0.05 (healthy versus RDA)
	Cholesterol -C(20,22,23)H_2_-	96059.4 ± 23169.5	48743.5 ± 13234.9	53844.7 ± 7671.8	<0.00001 (healthy versus LDA) <0.05 (healthy versus RDA)
	Cholesteryl ester -C(3)H	18006.6 ± 4574.6	7914.7 ± 2524.0	9110.7 ± 1023.9	<0.000001 (healthy versus LDA) <0.05 (healthy versus RDA)
	Free cholesterol C(3)-H	8083.6 ± 1364.7	5712.1 ± 2134.8	6336.3 ± 896.8	<0.05 (healthy versus LDA)
	Fatty acid -CH=CH-CH_2_-CH=CH-	17792.4 ± 3499.8	16022.5 ± 6185.2	14097.5 ± 7392.1	
	Fatty acid -(CH_2_)n-	1615749.3 ± 254434.4	1233196.9 ± 171325.7	1173938.0 ± 83905.6	<0.0001 (healthy versus LDA) <0.001 (healthy versus RDA)
	Fatty acid -CH_3_	142111.4 ± 43363.0	80141.0 ± 18795.6	88449.1 ± 11524.3	<0.00001 (healthy versus LDA) <0.05 (healthy versus RDA)
	Fatty acid -CH_2_-CO	56704.1 ± 13705.7	28166.0 ± 5875.4	29411.3 ± 2962.7	<0.0000001 (healthy versus LDA) <0.001 (healthy versus RDA)
	Fatty acid = CH_CH2_CH_2_	81686.8 ± 24564.3	34361.4 ± 11049.9	30264.7 ± 6366.5	<0.0000001 (healthy versus LDA) <0.001 (healthy versus RDA)
	Glycerol backbone –(CH_2_)-	2870.3 ± 665.6	1761.0 ± 404.6	1337.1 ± 235.6	<0.000001 (healthy versus LDA) <0.0001 (healthy versus RDA)
	Phosphoglycerides -CH-	13212.6 ± 2528.7	8006.1 ± 1844.0	5869.4 ± 1368.8	<0.000001 (healthy versus LDA) <0.0001 (healthy versus RDA)
	Phospholipids N(CH_3_)_3_	169112.5 ± 33440.1	107897.8 ± 23962.2	93096.8 ± 14665.1	<0.000001 (healthy versus LDA) <0.0001 (healthy versus RDA)
	Polyunsatured fatty acids (18:2, bis allylic protons)	57311.7 ± 18174.2	25810.8 ± 7702.0	21921.8 ± 4081.7	<0.0000001 (healthy versus LDA) <0.001 (healthy versus RDA)
	Sphingomyelin -CH = CH-	2997.6 ± 513.0	2024.2 ± 522.9	2359.6 ± 358.0	<0.05 (healthy versus LDA)
	Unsaturated fatty acid -CH = CH-	185955.1 ± 52747.7	117222.1 ± 28036.2	109687.6 ± 20646.2	<0.00001 (healthy versus LDA) <0.05 (healthy versus RDA)
	Unknow lipid	719.2 ± 85.1	872.8 ± 171.5	709.8 ± 73.7	<0.05 (healthy versus LDA)
**Liver water soluble (LWS)**	2-aminobutyrate	84.6 ± 35.1	105.7 ± 55.1	84.3 ± 37.0	
	2-hydroxybutyrate	39.1 ± 13.4	57.1 ± 23.1	58.5 ± 10.7	<0.05 (healthy versus LDA) <0.05 (healthy versus RDA)
	3-hydroxybutyrate	649.8 ± 210.0	638.5 ± 431.6	321.4 ± 235.7	
	2-hydroxyvalerate	192.8 ± 75.2	113.2 ± 76.0	206.4 ± 100.1	
	acetate	156.7 ± 54.5	99.7 ± 38.6	202.5 ± 52.1	<0.05 (healthy versus LDA) <0.05 (LDA versus RDA)
	Alanine	1684.8 ± 317.7	1251.5 ± 335.9	901.6 ± 486.6	
	Aspartate	223.3 ± 45.0	137.5 ± 40.5	117.9 ± 66.5	<0.05 (healthy versus LDA)
	Choline	5494.4 ± 1219.7	6712.6 ± 1175.0	6621.9 ± 2257.1	
	Creatine	613.5 ± 223.1	663.8 ± 331.4	1152.4 ± 298.8	<0.05 (healthy versus RDA) <0.05 (LDA versus RDA)
	Creatinine	429.1 ± 107.8	463.8 ± 182.7	401.9 ± 153.6	
	Formate	42.7 ± 17.4	51.0 ± 19.8	85.2 ± 64.0	
	Fumarate	51.7 ± 21.6	67.5 ± 18.4	47.6 ± 46.0	
	Glycerol	335.5 ± 78.1	678.9 ± 93.9	638.2 ± 201.4	<0.000000001 (healthy versus LDA) <0.05 (healthy versus RDA)
	sn-Glycero-3-phosphocoline	6496.9 ± 1722.2	5391.4 ± 1085.4	3154.2 ± 1317.5	<0.05 (healthy versus LDA) <0.001 (LDA versus RDA)
	Glycine	1180.1 ± 275.8	2091.9 ± 306.7	1759.6 ± 543.4	<0.0001 (healthy versus LDA)
	Glucose	316.3 ± 144.4	25.7 ± 25.7	3.1 ± 3.1	<0.0001 (healthy versus LDA) <0.001 (healthy versus RDA)
	Glutamate	91.8 ± 26.9	116.5 ± 35.7	73.8 ± 59.5	
	Isoleucine	43.3 ± 19.9	31.6 ± 11.8	35.0 ± 7.5	
	Isopropanol	16.5 ± 6.4	19.2 ± 8.7	37.3 ± 10.6	<0.05 (healthy versus RDA) <0.05 (LDA versus RDA)
	Lactate	977.6 ± 247.6	657.6 ± 120.0	837.5 ± 438.1	<0.05 (healthy versus LDA)
	Leucine	27.9 ± 27.9	44.0 ± 20.6	34.2 ± 15.3	
	Myoinositol	39.5 ± 16.9	91.2 ± 28.0	117.0 ± 35.0	<0.00001 (healthy versus LDA) <0.001 (healthy versus RDA)
	O-phosphocholine	1956.1 ± 474.6	2171.6 ± 548.4	3725.5 ± 1229.3	
	Succinate	236.6 ± 42.7	223.0 ± 31.8	193.7 ± 62.6	
	Uracil	52.7 ± 46.5	56.5 ± 37.3	71.1 ± 46.3	
	Uridine	69.3 ± 30.1	125.3 ± 22.6	113.3 ± 39.7	<0.00001 (healthy versus LDA)
	Unknow	278.6 ± 78.2	122.3 ± 37.7	82.6 ± 65.2	<0.05 (healthy versus LDA) <0.05 (healthy versus RDA)
	Valine	111.4 ± 36.9	78.1 ± 23.3	82.3 ± 29.4	
**Liver lipid soluble (LLS)**	Free cholesterol -C(3)H-	873.5 ± 393.6	1112.8 ± 395.5	1205.4 ± 349.2	
	Total cholesterol -C(18)H_3_	3367.7 ± 818.9	4317.6 ± 1699.1	4667.2 ± 686.1	<0.05 (healthy versus RDA)
	Multiple cholesterol protons	4930.3 ± 373.3	6027.2 ± 1271.3	6327.8 ± 567.6	<0.05 (healthy versus LDA) <0.05(healthy versus RDA)
	Fatty acids -CH_3_-(CH_2_)*_n_*-	78874.6 ± 7424.3	130014.7 ± 32490.4	97446.8 ± 9668.3	<0.0000001 (healthy versus LDA) <0.05 (healthy versus RDA)
	Fatty acids –(CH_2_)*_n_*-	672145.9 ± 75460.2	1034204.0 ± 270216.9	853648.9 ± 106179.1	<0.000001 (healthy versus LDA) <0.05 (healthy versus RDA)
	Fatty acids -CH_2_-CH_2_-CO	455655.5 ± 113667.9	549820.1 ± 115872.8	601105.4 ± 43428.1	
	Fatty acids -CH_2_-CH=	34891.7 ± 10498.9	57371.2 ± 39746.7	52710.2 ± 16408.1	<0.05 (healthy versus LDA)
	Fatty acids -CH_2_-CO	47047.6 ± 10198.8	79889.6 ± 33886.7	64893.3 ± 13046.1	<0.001 (healthy versus LDA) <0.05 (healthy versus RDA)
	Fatty acids = CH-CH_2_-CH=	26475.0 ± 10383.5	30851.1 ± 15070.1	24588.6 ± 4016.0	
	Fatty acids -CH = CH-	47835.0 ± 17628.7	78192.9 ± 42839.6	56367.3 ± 8661.7	
	Glycerol backbone –(CH_2_)-	199.2 ± 199.2	9267.3 ± 9212.6	1655.4 ± 1438.7	<0.001 (healthy versus LDA)
	Phosphoglycerides -CH-	13916.0 ± 4406.2	13895.6 ± 5307.9	16490.1 ± 3156.4	
	Phosphatidylcholine	12167.2 ± 4147.0	9516.6 ± 3512.6	12207.6 ± 3018.5	
	Sphingomyelin-choline	40154.9 ± 13163.9	29159.9 ± 11177.4	38578.1 ± 9073.0	
	Unknow 1	949.2 ± 156.8	581.2 ± 68.1	654.5 ± 82.1	<0.000001 (healthy versus LDA) <0.05 (healthy versus RDA)
	Unknow 2	3851.5 ± 629.2	3129.0 ± 318.4	3089.0 ± 365.6	<0.05 (healthy versus LDA)
**Urine water soluble (UWS)**	3-hydroxybutyrate	2057.4 ± 675.8	3756.0 ± 3329.4	1168.0 ± 895.7	<0.05 (healthy versus LDA) <0.05 (LDA versus RDA)
	2-hydroxy-3-methyl-valerate	1526.9 ± 419.4	2988.3 ± 1348.7	1687.1 ± 409.5	<0.05 (healthy versus LDA)
	4-hydroxypheny lacetate	572.2 ± 60.0	388.4 ± 138.6	444.2 ± 183.7	<0.001 (healthy versus LDA)
	acetate	6830.7 ± 2345.6	1482.6 ± 1266.6	4481.2 ± 4150.3	<0.001 (healthy versus LDA)
	Alanine	336.6 ± 108.1	417.8 ± 379.1	243.7 ± 52.1	
	Allantoin	349.2 ± 61.7	5061.5 ± 3542.5	5761.6 ± 5104.0	<0.000001 (healthy versus LDA) <0.05 (healthy versus RDA)
	Benzoate	22.8 ± 22.8	212.6 ± 212.6	0.8 ± 0.8	<0.05 (healthy versus LDA)
	Citrate	4678.6 ± 3277.1	1507.7 ± 1286.6	1985.2 ± 1733.5	<0.001 (healthy versus LDA)
	Choline	51.9 ± 51.9	365.0 ± 200.3	389.0 ± 89.4	<0.000001 (healthy versus LDA) <0.001 (healthy versus RDA)
	Creatine	8791.9 ± 2611.7	21502.2 ± 8430.5	12218.0 ± 9821.0	<0.0000001 (healthy versus LDA)
	Creatinine	10687.5 ± 2748.9	16234.4 ± 6726.3	23289.9 ± 10904.7	<0.0001 (healthy versus LDA) <0.001 (healthy versus RDA)
	Dimethylamine	27.9 ± 18.2	2248.5 ± 1265.4	2272.1 ± 1613.8	<0.00000001 (healthy versus LDA) <0.05 (healthy versus RDA)
	Dimethyl sulfone	1348.3 ± 300.8	275.6 ± 196.8	484.4 ± 220.2	<0.0000001 (healthy versus LDA) <0.05 (healthy versus RDA)
	Formate	1068.1 ± 143.3	231.5 ± 182.6	1282.3 ± 748.7	<0.00001 (healthy versus LDA) <0.05 (LDA versus RDA)
	Fumarate	29.1 ± 26.9	33.2 ± 22.1	26.2 ± 12.5	
	Glyoxilate	49.8 ± 28.7	21.0 ± 20.7	49.3 ± 48.6	<0.05 (healthy versus LDA)
	Hippurate	95714.3 ± 5107.3	24948.8 ± 14254.0	26049.2 ± 7194.1	<0.000000000001 (healthy versus LDA) <0.00001 (healthy versus RDA)
	Isobutyrate	347.6 ± 207.2	637.1 ± 346.9	333.2 ± 258.0	<0.05 (healthy versus LDA)
	Lactate	1473.5 ± 1473.5	5027.5 ± 4525.9	360.1 ± 151.7	<0.05 (healthy versus LDA) <0.05 (healthy versus RDA)
	Lactose	360.1 ± 151.7	1704.5 ± 1254.4	225.4 ± 225.4	<0.001 (healthy verus LDA) <0.05 (RDA versus LDA)
	N-phenylacetylglycine	18749.8 ± 4876.8	16422.8 ± 4249.5	15927.5 ± 2047.1	
	3-hydroxybutyrate	2352.4 ± 1941.6	1657.9 ± 1253.0	1903.9 ± 1513.0	
	Trimethylamine N-oxide	40396.6 ± 3423.7	11389.4 ± 6067.4	9964.5 ± 3920.7	<0.0000001 (healthy versus LDA) <0.05 (healthy versus RDA)
	Tyrosine	567.0 ± 125.3	145.2 ± 145.2	497.0 ± 474.1	<0.05 (healthy versus LDA)
	Unknow 1	7163.5 ± 1254.9	9895.8 ± 3864.0	10944.8 ± 3811.2	<0.05 (healthy versus LDA) <0.05 (healthy versus RDA)
	Unknow 2	388.2 ± 123.1	1715.2 ± 758.2	539.8 ± 167.9	<0.00001 (healthy versus LDA) <0.05 (LDA versus RDA)
	Unknow 3	643.9 ± 217.9	1255.1 ± 575.8	670.1 ± 163.3	<0.05 (LDA versus RDA)
**Urine lipid soluble (ULS)**	Total cholesterol C(18)H_3_	171.8 ± 89.8	53.6 ± 53.6	0.01 ± 0.01	
	Glycerol backbone –(CH_2_)-	18.4 ± 18.4	197.6 ± 42.3	165.3 ± 37.2	<0.0000001 (healthy versus LDA) <0.001 (healthy versus RDA)
	Fatty acids -CH_3_-(CH_2_)*_n_*-	122298.2 ± 1434.7	9413.9 ± 806.0	14060.8 ± 351.8	<0.05 (healthy versus RDA) <0.0001 (RDA versus LDA)
	Fatty acids -(CH_2_)*_n_*	85753.5 ± 12290.1	56153.8 ± 6561.3	94974.8 ± 7035.4	<0.001 (RDA versus LDA)
	Fatty acids -CH_2_-CH_2_-CO	274637.5 ± 36310.8	214963.2 ± 26967.3	217487.8 ± 24605.4	<0.0001 (healthy versus LDA) <0.05 (healthy versus RDA)
	Fatty acids = CH-CH_2_-CH=	140.6 ± 73.5	51.6 ± 51.6	96.8 ± 17.8	<0.05 (healthy versus LDA)
	Fatty acids -CH = CH-	643.0 ± 260.8	384.6 ± 189.4	325.7 ± 290.0	
	Unknow	1249.2 ± 93.4	1142.7 ± 126.7	983.2 ± 127.8	<0.05 (healthy versus LDA) <0.001 (healthy versus RDA) <0.05 (LDA versus RDA)

*p* values adjusted for false discovery rate are reported only for metabolites that differ significantly (*p* value <0.05) among the various comparisons performed. In particular, for each type of sample, healthy cows were compared to subjects with both LDA and RDA. To highlight metabolites that are significantly different between the two types of displacements, the comparison between cows with left and right abomasum dislocation was performed too.

From the analysis of serum samples, it appeared that healthy cows showed higher levels of hippuric acid, glycine, citrate, trimethylamine N-oxide, tyrosine, propionate, 2-aminobutyric acid, acetate, isoleucine, and alanine both when they were compared to LDA and RDA. In particular, hippuric acid is very high in healthy in comparison with LDA, instead, a high level of glycine was reported for healthy when compared to RDA. Furthermore, healthy cows showed higher levels of lipids respect to the diseased animals.

Diseased cows (with left or right displacement) compared with healthy animals appeared to be richer in 2-hydroxybutyrate, inosine, and lactate. Furthermore, glycine and 2-aminobutyrate were significantly higher for LDA than for RDA cows. Serum and liver contained high concentrations of 2-hydroxybutyrate in diseased cows compared with those from healthy ones.

In addition, the liver of diseased cows showed higher levels of myoinositol. Instead, healthy animals appeared to have higher levels of glucose in their liver and glycerol phosphocholine appeared to be higher in LDA subjects when compared to RDA animals.

Interesting information arises also from the analysis of liver lipids. Indeed, it resulted in that signal of glycerol backbone protons –(CH_2_)– was very high in cows with LDA when compared to healthy, but not in the case of the comparison with RDA animals. Then, signals of fatty acids arising from –(CH_2_)*_n_*, CH_3_(CH_2_)*_n_* and -CH_2_-CO protons were higher in diseased both when we considered LDA and RDA groups related to healthy.

At the end, from urine samples, high levels of hippuric acid and trimethylamine N-oxide in healthy subjects were confirmed and high levels of dimethylamine, choline, and creatinine resulted in diseased animals (both LDA and RDA). Furthermore, cows with LDA showed a very high level of 2-hydroxy-3-methyl valerate when compared to healthy animals; instead, cows with RDA have a very high level of dimethylamine.

The analysis of urine lipids is less straightforward because of the reduced number of lipids in urine, but among the eight lipophilic assigned fractions, it resulted that signals corresponding to glycerol backbone protons –(CH_2_)– were higher for diseased animals, as described for liver samples. Signals arising from –(CH_2_)*_n_*, CH_3_(CH_2_)*_n_* protons of fatty acids are higher in RDA group when compared to LDA subjects.

Summarizing the most significant findings obtained from the analysis of the three different matrices (serum, liver and urine samples), it is obvious that hippuric acid, citrate, dimethyl sulfone, trimethylamine oxide, tyrosine, propionate and 2-aminobutyric acid concentrations were lower (adjusted *p* values <0.01) in serum in both LDA and RDA cows. In addition, liver glucose concentration was very low (adjusted *p* value <0.001) in both LDA and RDA cows as compared to controls. Finally, only urine trimethylamine oxide concentration was (adjusted *p* value <0.05) lower for both right and left abomasal displacements. Moreover, phosphoglycerides, phospholipids, unsaturated and polyunsaturated fatty acids and sphingomyelin were lower (adjusted *p* values <0.05) in diseased cows' serum only. Finally, the biochemical network and pathway mappings performed on serum metabolites highlighted the “valine, leucine and isoleucine biosynthesis” and the “phenylalanine, tyrosine and tryptophan biosynthesis” as the most probable altered metabolic pathway in DA condition (see Supplementary Figures S1 and S2).

## 4. Discussion

Displaced abomasum was evaluated more comprehensively using serum, urine and liver metabolome of dairy cows in the present study.

The most commonly used indicators of energy status are NEFA and BHBA concentrations during both the dry and puerperal periods, as well as total protein and albumin during the puerperal period (Puppel and Kuczyńska 2016). A predictive association of elevated concentrations of BHBA with the risk of DA has been reinforced. Furthermore, low Ca and K concentrations, and high apo B100 concentration as well as, AST and GGT activities are related to the subsequent occurrence of DA (Sevinc et al. 2002; Civelek et al. 2006; Sen et al. 2006; Seifi et al. 2011; Constable et al. 2013). While serum and urine lactate levels from metabolomic analysis were increased in diseased groups, liver lactate was decreased in LDA cows. The timing, magnitude, and duration of peripartum increases in circulating concentrations of NEFA and BHBA are associated with the risk of the displaced abomasum, uterine disease, and reproductive performance from 1 through 20 weeks later (LeBlanc 2010). The generally used cut-off value for the diagnosis of subclinical ketosis is ≥1200 and up to 1400 μmol/L of blood BHBA (Suthar et al. 2013). Clinical ketosis is generally characterized by concentrations of BHBA in the blood >3000 μmol/L (Oetzel 2007). The different metabolites in cows with milk fever reflected the pathological features of negative energy balance and fat mobilization (Sun et al. 2014). In accordance with the above references, increased lactate, NEFA, BHBA, but decreased K levels in diseased groups were observed in the present study. Although some cows with LDA and RDA had also concomitant ketosis, BHBA level (1.94 ± 1.35) was significantly increased in LDA group as well as 3-hydroxybutyrate in serum and urine samples of LDA cows were increased. The 3-hydroxybutyrate was higher in RDA group compared with LDA cows. Increased blood concentrations of isopropanol are observed in ketotic cows (Sato 2009). In the present study, isopropanol and acetate was found higher in liver samples of RDA group than in healthy and LDA cows. Increased serum amyloid A and haptoglobin were found in cows with LDA or RDA/AV. Such an increase may indicate the presence of hepatic lipidosis in cattle with DA (Guzelbektes et al. 2010). The highest values of CRP and haptoglobin were observed in cows during the first month after calving (Dębski et al. 2016). In the present study, the highest values of CRP was observed in RDA cows. This may be attributed to fatty liver in cows with DA.

In ruminants, the principal gluconeogenic substrates include propionic acid, lactic acid, glycerol, and gluconeogenic amino acids (alanine, asparagine, arginine, aspartic acid, cysteine, glycine, histidine, methionine, proline, propionic acid, serine, valine). A lack of gluconeogenic substrates is an important risk factor in the pathogenesis of ketosis. Ketogenic amino acids (e.g., leucine and lysine) can also enter the tricarboxylic acid cycle by oxidative deamination. A disturbance in the tricarboxylic acid cycle may contribute to clinical ketosis (Sun et al. 2014). A non-metabolomic approach showed that plasma from LDA cattle exhibited significantly higher free fatty acid and BHBA, lower glucogenic amino acids, such as methionine, alanine, and serine, and higher ratio of ketogenic amino acids among blood free amino acids such as leucine and lysine (Hamana et al. 2010). In the present study, both gluconeogenic and ketogenic amino acids such as phenylalanine, isoleucine, and threonine were found decreased as well as ketogenic (leucine, lysine) and gluconeogenic (alanine, isoleucine, glycine, glutamine, proline, valine) amino acids in diseased cows. Lysine and glycine, and even leucine were lower in RDA cows. Ketosis in cows with LDA may be attributed to a lack of gluconeogenic substrates (propionic acid, glycerol, and gluconeogenic acids) and ketotic cows’ consummation of large amounts of ketogenic substances.

Carnitine transports the activated fatty acids from the cytosol into the mitochondrion via their corresponding carnitine ester (Stanley et al. 1992). Acetylcarnitine, as the shortest acylcarnitine, facilitates the movement of acetyl-CoA into the matrices of the mitochondria. Furthermore, in the mitochondria, carnitine acetyl-CoA transferase catalyzes the conversion of acetyl-CoA to C2, a membrane permeable metabolite which facilitates mitochondrial efflux of excess acetyl-CoA. Increased hepatic carnitine concentrations observed in 1 wk postpartum and might be regarded as a physiologic means to provide liver cells with sufficient carnitine required for transport of excessive amounts of NEFA during a negative energy balance (Schlegel et al. 2012). Especially the role of specific glycerophospholipids, sphingolipids, and acylcarnitines as potential biomarkers should be considered for metabolic adaptation of transition dairy cows (Kenéz et al. 2016). In the current study, serum carnitine level in diseased cows (especially in LDA group) was higher than in a healthy group. This may point to an increase in the mitochondrial oxidation of the ketogenic and gluconeogenic amino acids.

Valine, leucine, and isoleucine biosynthesis plays a vital role in milk protein synthesis. Hippuric acid, nicotinamide, and pelargonic acid are milk protein biomarkers (Wu et al. 2018). Hippuric acid, as an aromatic compound, could be converted into aromatic amino acids such as tryptophan, tyrosine, and phenylalanine, then be transformed into nicotinamide. Higher serum hippuric acid might also indicate more energy supplied by glucose metabolism and hormone regulation (Pero 2010). Urinary hippuric acid might reflect the dietary composition, hepatic function, disease state, and even metabolism alterations. A higher urinary hippuric acid level leads to more nitrogen loss and lower milk protein yield in cows fed low-quality forages, which supports the hypothesis that hippuric acid as excretory products is mainly produced from dietary protein degradation (Sun et al. 2016). In this present study, hippuric acid was one of the most prominent metabolites. Its lower level in serum and urine of diseased groups (especially in LDA group) together with lower valine, leucine, and isoleucine in serum may indicate less energy supplied and less nitrogen loss in a cow with DA.

Leucine, one of the three branch chain amino acids, acts as a signaling molecule in the regulation of overall amino acid and protein metabolism. Leucine is also considered to be a potent stimulus for the secretion of insulin from pancreatic β-cells (Sadri et al. 2017). The three branched-chain amino acids, leucine, isoleucine, and valine that are classified as essential amino acids, play important roles in regulating overall amino acids and protein metabolism. Marczuk et al. (2018) found that higher concentrations of glutamine, glutamic acid, isoleucine, and tyrosine in cows with primary ketosis, and that significant decrease in the concentrations of asparagine, histidine, methionine, and serine, alanine, leucine, lysine, and proline. In the current study, significant decreases in leucine, isoleucine, tyrosine, trimethylamine N-oxide, and valine of diseased groups were observed. Thus, protein and amino acid metabolisms seem to be adversely affected in the present study.

According to Shibano et al. (2005), the improvement of the glycine/alanine ratio in high producing dairy cows may indicate increased milk production and milk quality. Furthermore, the glycine/alanine ratio may be a useful indicator for determining nutritional deficiency from the transition period to the peak lactation period. Measurement of glycine/alanine ratio in serum may be useful for evaluating the nutritional status of a periparturient dairy cow. In the present study, this ratio was found much more impairment in the RDA group, suggesting their poor nutritional status.

The biopsy of the liver is the most reliable method for accurate estimation of the degree of fatty infiltration. It can be used to determine the concentration of triglycerides and the severity of the fatty liver (Herdt et al. 1983). Due to the rapid development of metabolomics in recent years, the use of this approach for disease biomarker assessment has become popular. A metabolomic approach revealed the primary differences including increases in BHBA, acetone, glycine, valine, trimethylamine-*N*-oxide, citrulline, and isobutyrate, and decreases in alanine, asparagine in plasma samples of cows with fatty liver (Xu et al. 2016). A study evaluating alterations of the lipid metabolome in dairy cows experiencing excessive lipolysis early postpartum showed that overall, excessive lipolysis in the high group came along with impaired estimated insulin sensitivity and characteristic shifts in acylcarnitine, sphingomyelin, phosphatidylcholine, and lysophospholipid metabolome profiles compared to the low group. From the detected phosphatidylcholines mainly those with diacyl-residues showed differences among lipolysis groups. Furthermore, more than half of the detected sphingomyelins were increased in cows experiencing high lipomobilization. Additionally, strong differences in serum acylcarnitines were noticed among lipolysis groups (Humer et al. 2016). The concentration of triacylglycerides in plasma drops at the day of parturition whereas the plasma level of many phosphatidylcholines and sphingomyelins increases steadily during early lactation (Imhasly et al. 2015). The measurement of specific representatives of phosphatidylcholines in plasma may provide a novel diagnostic biomarker of fatty liver disease in dairy cows (Gerspach et al. 2017). Choline can be metabolized to several other products, including betaine, phosphatidylcholine, and acetylcholine, each with critical biological roles (Garcia et al. 2018). The abnormal decline of certain specific phosphatidylcholines and sphingomyelins could be regarded as a promising biomarker indicative of fatty liver disease (Artegoitia et al. 2014; Imhasly et al. 2015). In the current study, while a lower level of many fatty acid fractions and cholesterol were encountered in serum and urine samples, the similar metabolites tended to be higher in liver samples in diseased groups. Fatty acids CH_3_-(CH_2_)*_n_* and fatty acids -(CH_2_)*_n_* were lower in urine samples of LDA cows. Phosphoglycerides, phospholipids, and unsaturated and polyunsaturated fatty acids and sphingomyelin were found lower in diseased cows' serum samples. The similar decreases in liver samples were not significant. sn-Glycero-3-phosphocoline was lower in liver samples of RDA cows. In our previous study (Basoglu et al. 2014), where metabolites identified and quantified by NMR analysis only in plasma samples were valine, 3 β-hydroxybutyrate, alanine, glutamine, glutamate, and succinate. Among these parameters, succinate decreased significantly in cows with RDA. Pronounced findings between LDA and RDA groups included significant changes in glutamine, glutamate, and 3 β-hydroxybutyrate. This previous study has been extended by the current one where different metabolites mentioned above were measured in serum, urine, and liver; thus pathogenic mechanisms related to energy metabolism of the disease have been more comprehensible.

## 5. Conclusions

The integration of several pathophysiological aspects, for example, lipolysis, ketogenesis, and oxidative capacity in cows with DA, by combining gluconeogenic and ketogenic amino acids, fatty acids fractions and cholesterol, ketone bodies, choline products, and carnitine, will likely provide more information than the classical measurement of NEFA and BHBA. The metabolomic profile, in the present study, clearly revealed that cows with DA (especially with LDA) have been at risk ketosis and fatty liver and that analyzing changes of the serum, urine and liver metabolome and identifying new biomarkers by metabolomic approach can help to understand the multifaceted disease. The biochemical network and pathway mappings performed on serum metabolites highlight the ‘valine, leucine and isoleucine biosynthesis’ and the ‘phenylalanine, tyrosine and tryptophan biosynthesis’ as the most probable altered metabolic pathway in DA condition.

## Supplementary Material

Supplemental Material
