# Supplementary material for: Nuclear magnetic resonance (NMR)-based metabolome profile evaluation in dairy cows with and without displaced abomasum
Source: Vet Q. 2020 Jan 7;40(1):1–15. doi: 10.1080/01652176.2019.1707907 (PMC6968509; doi:10.1080/01652176.2019.1707907)
Supplement: Supplemental Material [file TVEQ_A_1707907_SM6259.docx]

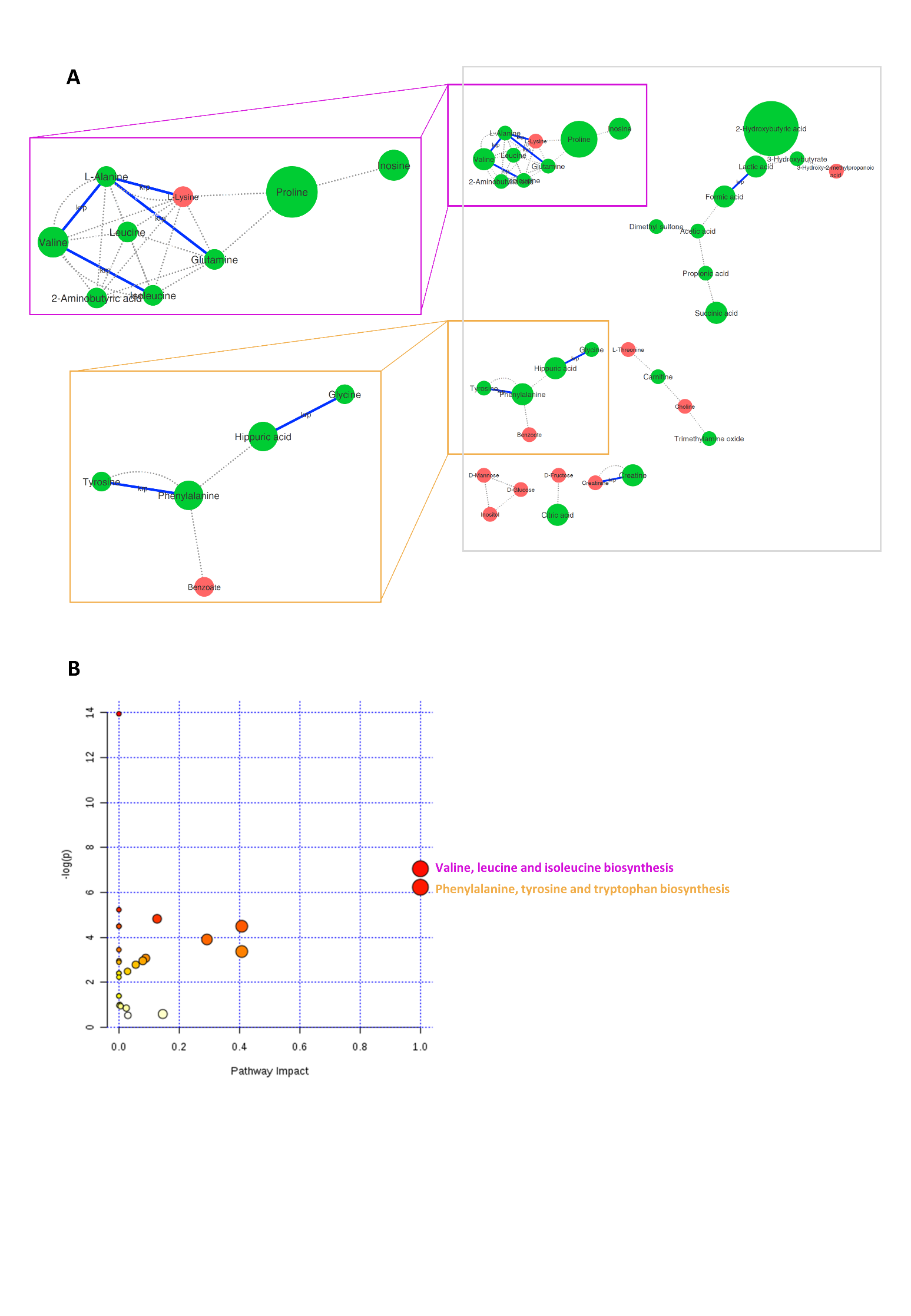
**Biochemical network mapping and pathway analysis**

**Figure S1.** Biochemical network mapping and related pathway analysis for serum water-soluble metabolites from the comparison between healthy and left displaced abomasum cows. The global network graph of metabolites (on the right-side of panel A of the figure) was obtained using the MetaMapp online tool^1^ where green nodes represent metabolites whose concentration is signiﬁcantly different (adjusted^2^ *P* value<0.05) in the comparison, while red nodes represent metabolites whose concentrations are not statistically relevant. Nodes size reflects Fold-Change values. Biochemical and chemical relationships among metabolites are represented by KRP (KEGG Reaction Pairs) and TMSIM (Tanimoto similarity) bold blues edges and black dashed links respectively. In panel B, the MetaboAnalyst^3^ pathway mapping is reported only for statistically significant metabolites (adjusted *P* values<0.05). In detail, each dot represent a specific metabolic pathways which is plotted depending on the “pathway impact” and related “-log(*p* values)”. The plot highlights as the most significant metabolic pathways for impact and -log(p values) the “valine, leucine and isoleucine biosynthesis” and the “phenylalanine, tyrosine and tryptophan biosynthesis” (reported on the right side of the plot) whose respective biochemical networks are enlarged in the magenta and orange boxes depicted on the left-side of panel A of the figure.


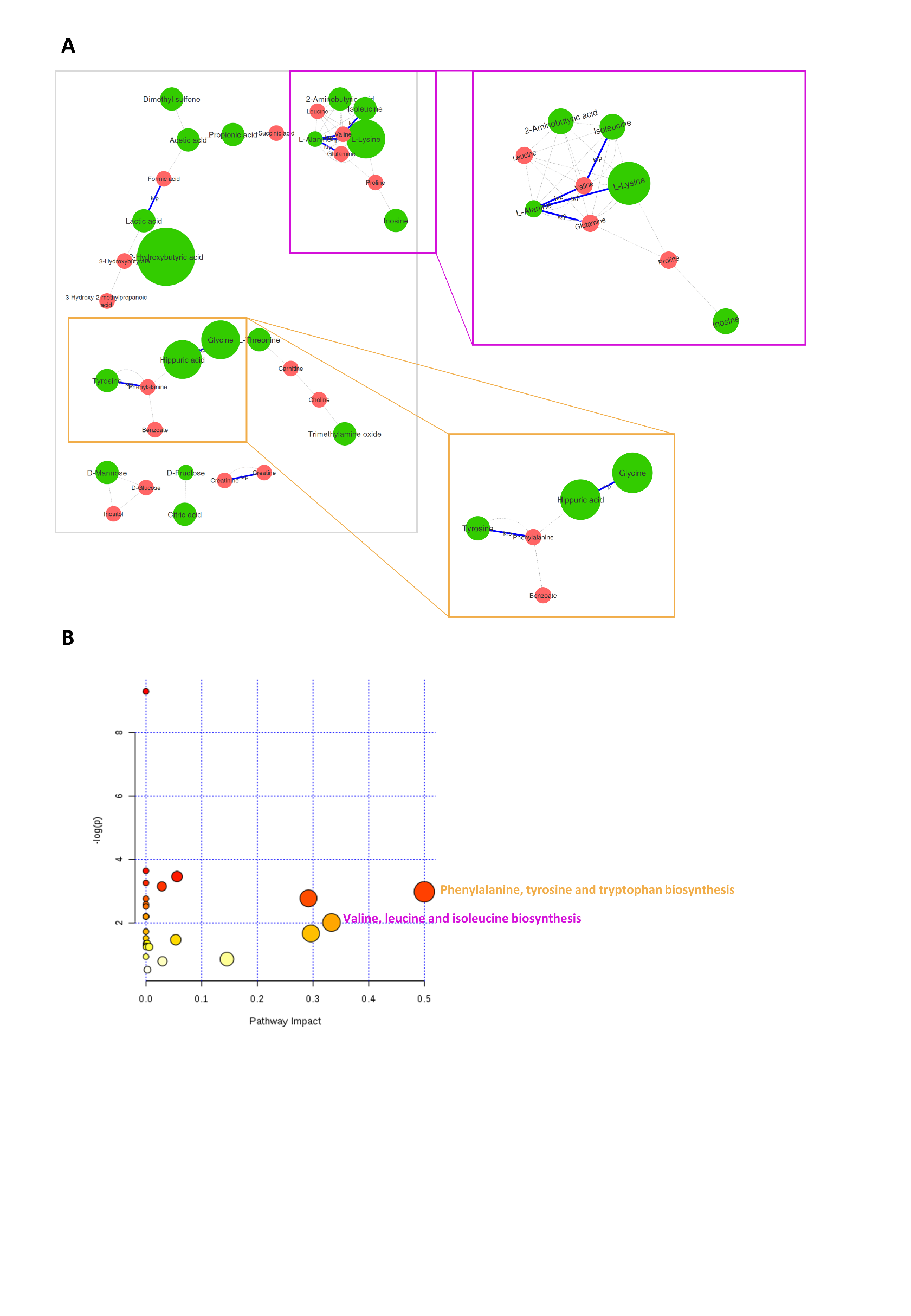


**Figure S2.** Biochemical network mapping and related pathway analysis for serum water-soluble metabolites from the comparison between healthy and right displaced abomasum cows. The global network graph of metabolites (on the left-side of panel A of the figure) was obtained using the MetaMapp online tool^1^ where green nodes represent metabolites whose concentration is signiﬁcantly different (adjusted^2^ *P* value<0.05) in the comparison, while red nodes represent metabolites whose concentrations are not statistically relevant. Nodes size reflects Fold-Change values. Biochemical and chemical relationships among metabolites are represented by KRP (KEGG Reaction Pairs) and TMSIM (Tanimoto similarity) bold blues edges and black dashed links respectively. In panel B, the MetaboAnalyst^3^ pathway mapping is reported only for statistically significant metabolites (adjusted *P* values<0.05). In detail, each dot represent a specific metabolic pathways which is plotted depending on the “pathway impact” and related “-log(p values)”. The plot highlights as the most significant metabolic pathways for impact and -log(p values) the “valine, leucine and isoleucine biosynthesis” and the “phenylalanine, tyrosine and tryptophan biosynthesis” (reported on the right side of the plot) whose respective biochemical networks are enlarged in the magenta and orange boxes depicted on the right-side of panel A of the figure.

**References**

1. Barupal, D. K. *et al.* MetaMapp: mapping and visualizing metabolomic data by integrating information from biochemical pathways and chemical and mass spectral similarity. *BMC Bioinformatics* **13**, 99 (2012).

2. Benjamini, Y. & Hochberg, Y. On the adaptive control of the false discovery rate in multiple testing with independent statistics. *J. Educ. Behav. Stat.* **25**, 60–83 (2000).

3. Chong, J. *et al.* MetaboAnalyst 4.0: towards more transparent and integrative metabolomics analysis. *Nucleic Acids Res.* **46**, W486–W494 (2018).
